# Prevalence of Candida auris Among High-Risk Patients at a Comprehensive Cancer Center

**DOI:** 10.1017/ash.2024.232

**Published:** 2024-09-16

**Authors:** Adina Feldman, Micah Bhatti, Jane Powell, Amy Spallone, Roy Chemaly

**Affiliations:** MD Anderson Cancer Center; The University of Texas MD Anderson Cancer Center

## Abstract

**Background:** Candida auris (C. auris) is a multidrug-resistant fungus that is increasingly implicated in outbreaks in healthcare facilities worldwide. The Centers for Disease Control and Prevention (CDC) and the Texas Department of State Health Services recommend healthcare facilities screen patients who are considered high-risk for C. auris, including patients with an overnight stay in a healthcare facility outside the United States (U.S.) in the previous year, or recently stayed in a rehabilitation (rehab) facility, long-term acute care (LTAC), or skilled nursing facility (SNF). Screening patients for C. auris colonization allows for early implementation of infection control measures, preventing transmission to healthcare workers and other patients. According to the CDC, most cases of C. auris result from local spread within and among healthcare facilities in the same city or state. In Texas, 160 clinical cases have been reported during the past 12 months. At present, the necessity of screening high-risk patients at our center for C. auris is not known. We aimed to determine the prevalence of C. auris colonization among our patient population. **Method:** During a 4-week period, we performed targeted screening of patients meeting the CDC’s high-risk definition for C. auris. Admitted patients were screened by an Infection Preventionist (IP) using the electronic health record to identify patients who were either international or admitted from a rehab or care facility. A composite swab of bilateral axilla and groin creases was collected using an eSwab™ (Becton Dickinson) and sent to a reference lab (Mayo Clinic Laboratories) for polymerase chain reaction (PCR)-based detection of C. auris. Additionally, we reviewed historic cases of C. auris diagnosed at our institution to better define our at-risk patients. **Results:** Between July 14 – August 8, 2023, we consecutively screened 25 high-risk patients, including 18 (72%) international and 7 (28%) patients from rehabs, LTAC, or SNF. None were positive for C. auris. Since 2019, we identified six patients with C. auris positive cultures, including five clinical cases and one colonization case. Five patients were international and one was local with no history of international travel or stay in a care facility. Interestingly, all six were known to be colonized with extended-spectrum beta-lactamase (ESBL) E. coli. **Conclusion:** We have a very low prevalence of C. auris among CDC-defined high-risk patients. A review of historic C. auris cases indicated an association with colonization by other multidrug-resistant organisms, specifically ESBL E. coli, which will inform future screening protocols at our institution.

**Disclosure:** Roy Chemaly: Contracted Research paid for my institution: Merck, Karius, AiCuris, Ansun Pharmaceuticals, Takeda, Genentech, Oxford Immunotec, and Eurofins-Viracor; Honorarium/Ad Board/Consultant: ADMA Biologics, Janssen, Merck/MSD, Partner Therapeutics, Takeda, Shinogi, AiCuris, Roche/Genentech, Astellas, Tether, Oxford Immunotec, Karius, Moderna, and Ansun Pharmaceuticals; Stock Options: Xenex

